# A vibration sensor approach to detect intra-articular needle tip placement in the knee joint: a proof-of-concept study

**DOI:** 10.1186/s12891-021-04836-y

**Published:** 2021-11-15

**Authors:** Rit Apinyankul, Kritsada Siriwattanasit, Kakanand Srungboonmee, Witchaporn Witayakom, Weerachai Kosuwon

**Affiliations:** 1 Department of Orthopedics, Faculty of Medicine, Khon Kaen University, Khon Kaen, 40002 Thailand; 2 Center of Data Mining and Biomedical Informatics, Faculty of Medical Technology, Mahidol University, Nakhonpathom, 73170 Thailand

**Keywords:** Intra-articular knee injection, Needle tip placement, Vibration sensor, Vibration frequency band power

## Abstract

**Background:**

Intra-articular injection in the dry knee joint is technically challenging particularly for the beginners. The aim of this study was to investigate the possible use of the vibration sensor to detect if the needle tip was at the knee intra-articular position by characterizing the frequency component of the vibration signal during empty syringe air injection.

**Methods:**

Two milliliters of air were injected supero-laterally at extra- and intra-articular positions of a cadaveric knee joint, using needles of size 18, 21 and 24 gauge (G). Ultrasonography was used to confirm the positions of needle tip. A piezoelectric accelerometer was mounted medially on the knee joint to collect the vibration signals which were analyzed to characterize the frequency components of the signals during injections.

**Results:**

The vibration frequency band power in the range of 500–1500 Hz was visually observed to potentially localize the needle tip placement during air injection whether they were at the knee extra-articular or intra-articular positions, as demonstrated by the higher band power (over − 40 dB or dB) for all the needle sizes. The differences of frequency band power between extra- and intra-articular positions were 18.1 dB, 26.4 dB and 39.2 dB for the needle size 18G, 21G and 24G respectively. The largest difference in spectral power was found in the smallest needle diameter (24G).

**Conclusions:**

A vibration sensor approach was preliminarily proved to distinguish the intra-articular from extra-articular needle placement in the knee joint. This study demonstrated a possible implementation of an alternative electronic device based on this technique to detect the intra-articular knee injection.

## Background

Intra-articular knee injection is a common procedure in treatment and diagnosis of the knee joint pathology. For example, local anesthesia injection to the knee joint is done in the elective knee arthroscopy cases [1, 2] and in some knee physical examinations that need to be done under anesthesia [3], gadolinium contrast media injection in knee joint imaging [4] and viscosupplement and corticosteroid injection to alleviate the knee pain. These procedures are done as the knee osteoarthritis is one of the most common degenerative joint disease and impacts functional capacity in elderly (prevalence of 3.8% and 2.7 times more common in women than men) [5]. Disease pathophysiology of knee osteoarthritis involves inflammatory process of synovium and structural changes of articular cartilage including subchondral bone [6]. Most of the patients suffer from pain, stiffness and impaired joint mobility and often require intra-articular injection with non-steroidal anti-inflammatory drug, corticosteroid or viscosupplement agent for pain relief and inflammation control to improve joint function.

The accuracy of intra-articular needle placement is critical to prevent soft tissue complication and achieve a good treatment outcome by avoiding inadequate analgesia and agent concentration in the intra-articular space. To minimize potential complications of extra-articular injection from local tissue damage, such as atrophy of muscle and subcutaneous fat, pain, skin hypo-pigmentation [7] and skin necrosis [8], proper placement of needle is advocated to confirm intra-articular needle position [9]. Many injection techniques with different knee postures are proposed for agent delivery to the knee, however the best portal and posture is still controversial [10]. A systematic review demonstrates that the superolateral approach in extended knee has the best accuracy around 91% compared to other approaches in the similar posture (lateral mid-patellar 85%, anteromedial 72% and anterolateral 67%) [11]. Additionally, in 90^o^-bended knee position, a study using squishing technique [12] and post-injection mini air-arthrography [13] demonstrates that injection with modified anterolateral approach has higher accuracy (89%) than the superolateral approach (58%) [14].

No matter what techniques used, the accuracy of needle tip placement is essential, particularly when the knee effusion is not present or the symptomatic dry joint [15]. The extra- or intra-articular position of needle tip can usually be detected by ultrasonography. The ultrasound-guided knee injection and aspiration offers greater accuracy and clinical improvement over using only the conventional landmark technique [16–19], however, the imaging technique requires extra time and the cost of machine prohibits its use in the limited-resource settings. Without an ultrasound machine, the tactile sensation, which is the sensing of tissue resistance felt at the performer’s hand, can generally be used to distinguish the needle tip positions in the clinical practice. The loss-of-resistance feeling or absence of the backflow when the needle tip in intra-articular, detecting from the tactile feedback felt at the syringe, has also been proposed [20] and implemented as an instrument [21] for the imaging-free technique to increase the intra-articular injection accuracy. However, the learning curve is steep for the technique, especially without the assistance of a device.

Instead of detecting the tissue resistance, this study proposed detecting the vibration of air injection to indicate the needle tip placement. The idea was based on the previous studies that the vibration sensor approach has been used to detect particle flow in the fluidized bed reactor. A fluidized bed reactor, used in many industrial applications, is a device that carries multiphase chemical reactions. Vibration signals, usually taken from the piezoelectric accelerometer mounting outside the fluidized bed reactor, can be used to non-invasively detect the interested particle flow by characterizing the vibration frequency components, for example, to distinguish sand-oil-water flow from sand-water flow [22] and to detect the sand flow in the gas-sand flow [23] by observing the power spectral density for particular frequency bands of the vibration signals. Specifically, the sand flow in different media (oil, water [22], gas) exhibits different vibration characteristics. The piezoelectric accelerometer is a surface contact sensor that converts the vibration signal in the form of acceleration of the piezoelectric material inside to the electrical analog signal that can usually be collected by an analog-to-digital system. After transforming those vibration signals into the time-frequency domain commonly by the short-time Fourier transform (STFT) and the wavelet transform [24], different patterns corresponding to the different flow phases, e.g. with or without solid particles in the flow, can be observed and characterized. The vibration detected in the multiphase flow in the fluidized bed reactor comes from the moving interaction between particles in the flow and reflects in the frequency characterization of vibration [24].

When different multiphase flows can be classified using the vibration frequency analysis, detection of injected air flow through different anatomical structures also seems possible. The flow of air injection originated from the needle tip at different anatomical locations might be distinguishable using vibration frequency analysis, suggesting the needle tip placement either intra- or extra-articular positions. The aim of this study was therefore to compare the vibration signals of intra-articular and extra-articular air injection into the cadaveric knee using the piezoelectric accelerometer sensors, in order to characterize the frequency power band of the detected vibration signal. The idea was to generate the vibration due to the flow of air injection and analyze the signal. Specifically, the vibration signals when the injected air flow through the tendon (extra-articular position) should be different from the injected air flow through the knee joint space (intra-articular position) for all the three needle sizes.

## Methods

### Experimental setup

**Fig. 1:**
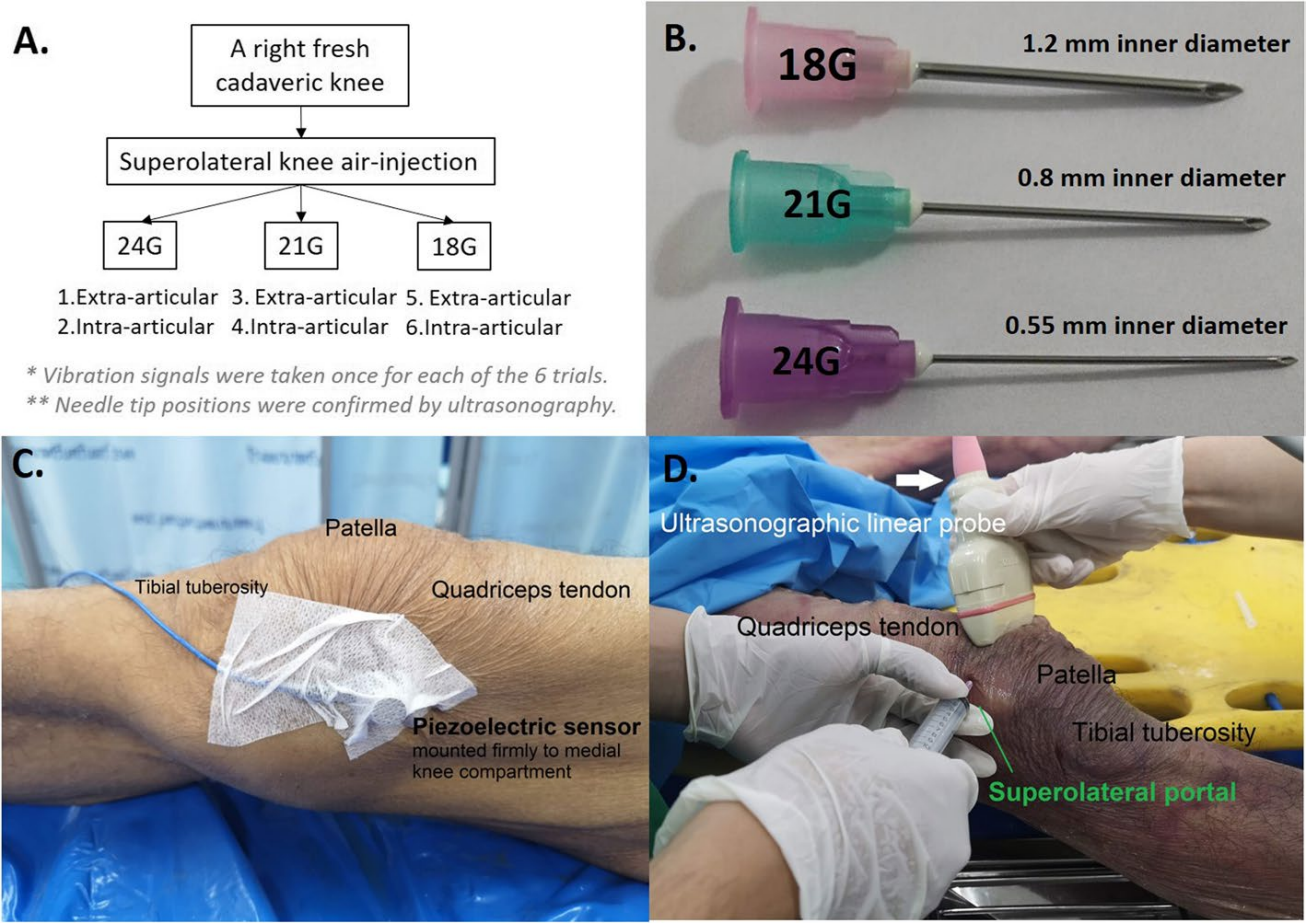
The workflow of the study consisted of vibration signals measurement for six consecutive trials of the knee injections (**A**). Three needle sizes were used (**B**). Experimental setup was done by mounting the accelerometer firmly on the medial collateral ligament just below the medial border of patella, about the mid-patellar level (**C**). An empty syringe was placed at supero-lateral knee, ready for the 2-ml air injection, and a linear ultrasound probe was positioned on the quadriceps tendon above the patella (**D**)

Six consecutive trials of air injection to the knee joint (three needle sizes at two needle tip placements) were done to collect the vibration signals (Fig. 1A). Three needles were the 1.5-in. (38-mm) thin wall needles of gauge size 18G (HN-1838ET), 21G (HN-2138-ET) and 24G (HN-2438-ET), Nipro needles, 3–9-3, Honjo-Nishi, Kita-ku, Osaka, Japan (Fig. 1B), all of them were attached to a 10-ml centric tip Nipro luer lock syringe (SY3-xLC-EC). The sequence of injection was 24G, 21G and 18G (smallest to largest diameter to minimize the skin leakage due to needle puncture) with the needle tip at extra-articular following by intra-articular position of each needle. One fresh 68-year-old male cadaver of 168-cm height without any musculoskeletal diseases, preserved at 4 degree Celsius for 48 h after the congestive heart failure death, was set up for the fully-extended right knee injections at room temperature. A surface-contacting piezoelectric accelerometer sensor (352C33, PCB Piezotronics, USA) was firmly mounted at medial compartment of the knee, approximately at the mid-patellar level on the medial collateral ligament which was just below medial border of patella (Fig. 1C). Ultrasonographic linear probe was used to obtain the ultrasound image in order to confirm whether the needle tip was at extra-articular (quadriceps tendon) or intra-articular (knee joint cavity) position (Fig. 1D). After the desired needle tip position was confirmed by the ultrasound image, two milliliters of the air were injected as fast as possible with superolateral approach from the empty syringe for all cases. The air-flow-induced vibration signals were collected through the sensor (see *Acquisition of the vibration signals* for details).

### Ultrasonographic confirmation of the needle tip positions

**Fig. 2:**
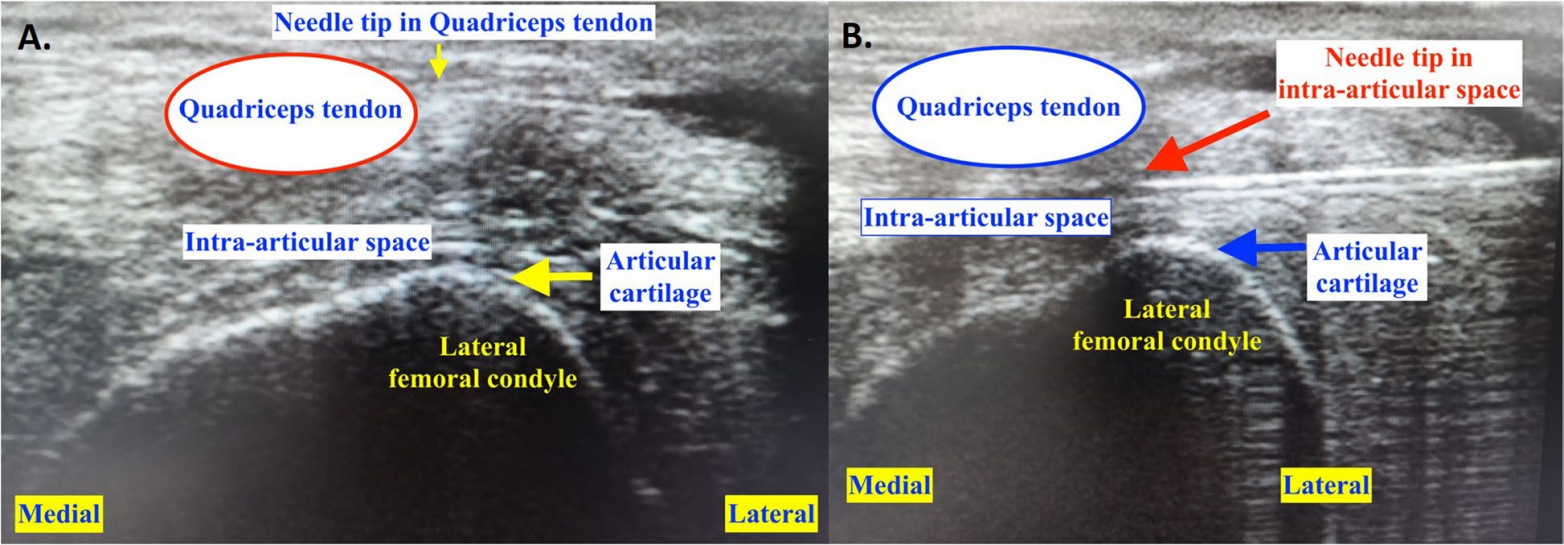
Ultrasonographic visualization of the needle tip to confirm extra-articular (**A**) and intra-articular (**B**) positions. Extra-articular position was defined as the needle tip was at the quadriceps tendon. For intra-articular position, the needle tip was in the intra-articular space beneath the quadriceps tendon and above the articular cartilage of lateral femoral condyle

The ultrasonographic linear transducer was used to check for anatomical landmarks of the knee joint. A skillful musculoskeletal radiologist confirmed the positions of needle tip in real-time by using Siemens S3000 ultrasonography machine and linear probe (14 L5), with a frequency between 5.0 and 14.0 MHz. The probe was placed on the quadriceps tendon, above the superior pole of the patella (Fig. 1D), and angled so that the quadriceps tendon and articular cartilage of lateral femoral condyle were seen (Fig. 2). The extra-articular position was defined as the needle tip was in the quadriceps tendon (Fig. 2A). The intra-articular position was identified by the space beneath quadriceps tendon and above articular cartilage of lateral femoral condyle (Fig. 2B). Air flow pulse after each injection was also observed from the ultrasound to ensure no needle block occurred during each injection.

### Acquisition of the vibration signals

**Fig. 3:**
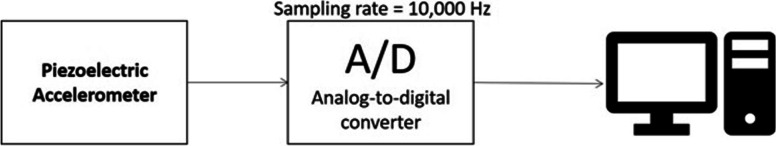
Schematic diagram of the vibration signal acquisition system. The system consisted of a piezoelectric accelerometer, an analog-to-digital converter with sampling rate of 10,000 Hz (Hz) and a computer to collect and display the signals

The vibration signals were collected from the piezoelectric accelerometer (352C33, PCB Piezotronics, USA). The signal was sampled at 10,000 Hz (Hz) through an analog-to-digital converter (DEWE-43-A, 24-bit resolution with anti-aliasing filter), connecting to a computer laptop with the data acquisition software (Dewesoft X) to record the data (Fig. 3). The sensitivity of accelerometer was 102.9 mV/gravitational acceleration (mV/g). The vibration signals were recorded in the unit of gravitational acceleration (g) for 3 s. No other filter was applied to the signals.

### Signal processing and parameters calculation

The vibration signals were analyzed in time-frequency domain without additional filter. The short-time Fourier transform was applied to the digital time-domain signal in order to visualize the power spectral density of the signal as time progressing, i.e. the spectrogram. Interested frequency band was determined by visual inspection. Quantification of the frequency band power was done by calculating the summation of the spectral power of the signals in the interested frequency bands after the discrete Fourier transform (256 samples per segmentation with the Hamming window of 250 samples and the overlap of 256 samples) of the signals. All the signal processing was done in MATLAB R2019a (The MathWorks, MA, USA).

## Results

### Time frequency response

**Fig. 4:**
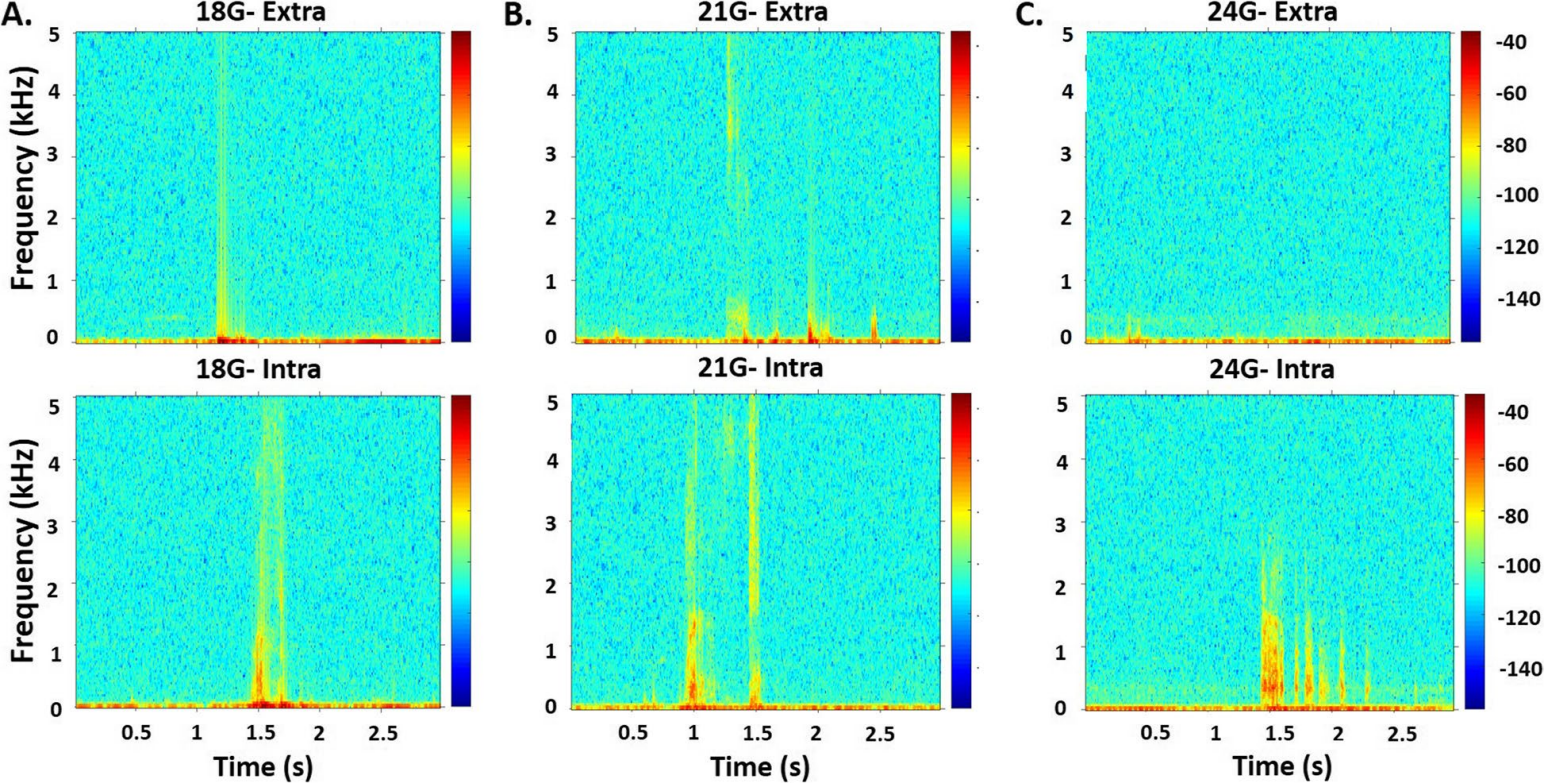
Spectrogram of the vibration signal illustrated frequency (kilohertz or kHz) and time in second (s) when the injection was done at the extra-articular (top row) and intra-articular (bottom row) knee joint for the needle gauge of 18G (**A**), 21G (**B**) and 24G (**C**). The color bars represent the corresponding power spectral density in decibel (dB)

The time-frequency responses are displayed in the spectrogram shown in Fig. 4. Higher power spectral density at the high frequency band (approximately 500–1500 Hz) was observed when the needle tips were in the knee intra-articular position (bottom row), but not in the extra-articular position (top row). The phenomenon was seen in all the needle sizes (Fig. 4A-C). Specifically, in the high frequency band, minimum vibration power during the extra-articular injection and maximum vibration power during the intra-articular injection were observed from the smallest diameter needle (24G) (Fig. 4C).

### Spectral power

**Fig. 5:**
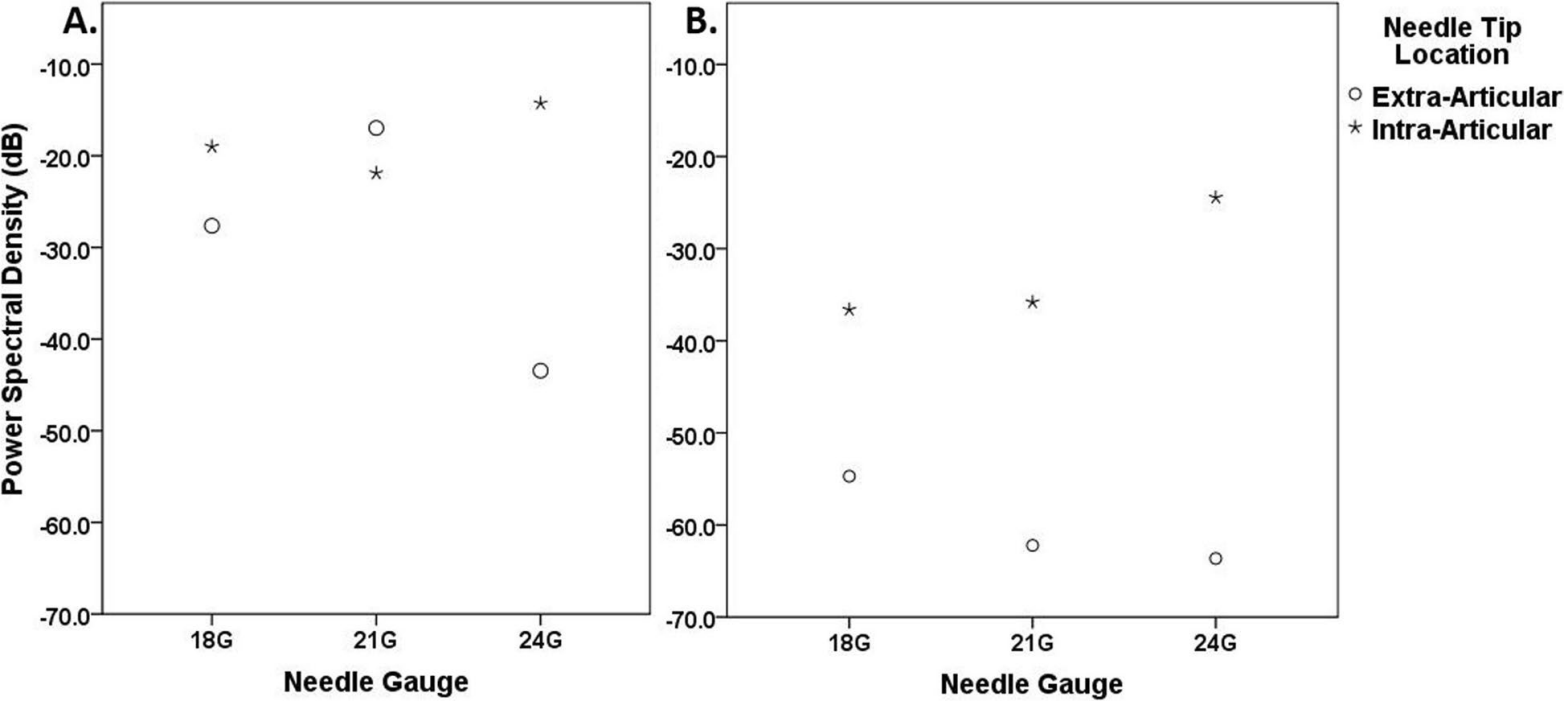
Power spectral density in decibel (dB) of the **A.** low (50–500 Hz) and **B.** high (500–1500 Hz) frequency bands for needle gauge of 18G, 21G and 24G, when the needle tips were either at the extra-articular (round markers) or intra-articular (star markers) locations

Summation of power spectral density over the interested frequency band (frequency band power) was used to quantify the spectral power. Figure 5 showed the summation of power spectral density in the low (50–500 Hz) and high (500–1500 Hz) frequency bands. In the low frequency band, the spectral power was high, approximately over − 40 dB (dB) (dark red color of the spectral power in Fig. 4), and it was hard to distinguish between the extra-articular or intra-articular needle tip locations (Fig. 5A). When the needle was intra-articular, higher power spectral density of the high frequency band was observed, compared to the case of the extra-articular needle positions (Fig. 5B). The difference in power spectral density was 18.1 dB, 26.4 dB and 39.2 dB for the needle size 18G, 21G and 24G respectively.

## Discussion

Accurate needle tip placement for the knee intra-articular injection is typically challenging for novice physicians especially in a dry knee joint compared to a knee joint with effusion in which the excess synovial fluid makes it easier to confirm the intra-articular position [20]. A fresh cadaver with dry knee joints was therefore used as the study model to prove the concept that the vibration sensor, mounted on the medial side of the knee, can enhance the accuracy of needle tip placement by distinguishing the vibration signal when the needle tip was at the extra- and intra-articular positions of the knee joint during the hard-push air injection using superolateral approach. In clinical practice, the needle tip position can be confirmed by imaging technique like ultrasonography [18, 19] or fluoroscopy [20] in which additional machine and the specialist are required to complete the procedure. The image-free approaches include detection of audible squishing sound [12] and feedback tactile sensation [21]. The detection of audible squishing sound during range of motion generated after the small amount of agent injection gives good sensitivity [12] but it could not be practical to move the knee with severe joint pain. The feedback tactile sensation techniques usually rely on the hand sensing such as the loss-of-resistance technique [14] and backflow detection [20], in which the sensing ability depends on the operator’s experience and can be challenging for the beginners. Consequently, the special equipment has been proposed to help detect the change in tissue resistance during injection to facilitate the learning of this procedure [21]. This study, therefore, proposed an alternative technique in detecting the vibration of the air injection to locate the needle tip position which can be used either as an equipment to confirm the needle tip placement like the imaging technique or as a feedback tool to practice the hand sensing ability.

The results have shown that the power spectral density in the high frequency band (500–1500 Hz) in Fig. 5B was more likely to distinguish between the extra- and intra-articular needle tip positions in all needle sizes, compared to the low frequency band (50–500 Hz) as seen in Fig. 5A. The spectral power in the low-frequency band was generally high for both positions of all the needle sizes (Fig. 5A). Specifically, the intra-articular air flow injection detected from an accelerometer placed at the medial compartment of the knee can therefore be characterized as the higher power spectral density (over − 40 dB) in the frequency band of 500–1500 Hz.

Characterizing the vibration signals from the air injection in the knee joint can be similar to the case of vibration monitoring in the fluidized bed from previous studies [22–24]. The distinct frequency components of vibration signals developed from the presence of particle flow is commonly observed in the phase flow in the fluidized bed where the particles velocities can be controlled and the phase flow including bubbles formation can be seen from the side glass window of the chamber. For example, large bubbles and particles-wall interaction usually correspond to low frequency signals [24]. Likewise, in the present study, the power of low frequency components could be due to the air flow that might fluctuate the peri-articular tissues in which it could result from when the injection was at either the extra- or intra-articular sites (Fig. 4). The high frequency signal is usually associated with the air flow [24]. Air flow velocity, bubble formation and bubble size also reflect in the different spectral power of vibration signal. When the air flow is present in the fluidized bed, sharp peak of the power spectrum at 1000 Hz is found regardless of the flow velocity. But when the air velocity is increasing, higher frequency components gradually appear between 3600 and 4000 Hz. When the frequency components in the range of 800–1500 Hz appears, it is the sign of bubble formation [24]. The range of 500–1500 Hz was selected accordingly [24] and based on the observation in Fig. 4 that quantification of this frequency band should be able to distinguish these two needle tip positions. Therefore, high frequency component observed should be explained as the air flow induced vibration in the joint cavity as seen when the needle tip was at intra-articular location during empty syringe air injection, (Figs. 4 and 5).

However, needle sizes may affect the power spectral density of the vibration signal. With the similar volume flow rate in the extra-articular cases, the needle with larger diameter can cause more fluctuation to the peri-articular tissues as in the case of the largest diameter needle (18G), where the power spectral density in the high frequency band was higher than other needle sizes (Fig. 5B). For the case of intra-articular 24G needle air injection, smaller cross-sectional flow causing faster flow velocity could also affect higher spectral power (Fig. 5B). Despite the flow velocity, higher spectral power in the high frequency band of the larger diameter needles (18G and 21G) could also be due to the moving interaction between the injected air particles and the accumulated air particles from the previous injection. In clinical practice, these three needle sizes are selected for the articular knee injection depending on operators’ preferences. The smaller diameter of the needle provides less injection pain to the patients but harder for the clinicians to feel the loss-of-resistance if the technique is solely used to locate the needle tip. An assisted device to enhance the needle tip injection accuracy could be useful for the beginners especially when using the smaller diameter needles. Further study with more samples and random sequence of injection is recommended to increase reliability.

Settings that were critical in this study were the location of sensor, the mounting technique and the external vibration. The vibration sensor should be mounted firmly to the flat surface with minimum impact load and external vibration. Relative movement between the sensor and the surface should also be careful as it could produce high spectral power. During injection from the lateral side of the knee, the aforementioned factors should be minimized when placing the sensor on the medial collateral ligament, adjacent to the joint space.

The use of one cadaveric knee sample with single injection per scenario was the major limitation of this study. This was due to the study was done during the COVID-19 pandemic when body donation was restricted. Study in human was possible despite that an advantage of cadaveric study was the absence of needle reflex that could provide the muscle twitch contributing as a small external impact load to the sensor. More cadaveric sample should be obtained after the pandemic. Moreover, there was an effort to minimize the air accumulation in tissue and joint space that could affect the vibration signal in the later trials. That was why the injection was done once in each scenario. Either larger number of cadaveric knees or smaller amount of the air at each injection could help increase number of trials in the future study.

## Conclusions

The superolateral empty syringe air injection technique with a vibration sensor on the medial collateral ligament at the mid-patellar level was proved to distinguish the intra-articular from extra-articular needle placement in the knee joint by considering the spectral power at the frequency band of 500–1500 Hz. This vibration sensor approach could be an alternative technique to detect the accurate location of needle placement. This study demonstrated the possible electronic device implementation of this technique to detect the intra-articular injection when ultrasound machine is not available.
